# Supplementary material for: Investment modeling for scalable agricultural learning
Source: PLoS One. 2026 Mar 10;21(3):e0343613. doi: 10.1371/journal.pone.0343613 (PMC12974861; doi:10.1371/journal.pone.0343613)
Supplement: S1 File — (DOCX) [file pone.0343613.s001.docx]

**Supplementary Methods: Estimation of Parameter *a***

To estimate the parameter *a* (the sharing or interaction rate), we developed and compared two dynamic models representing the spread of information in the learning initiative.

**Model 1: Simplified Linear Growth Model**

The first model describes the change in the number of informed individuals *I(t)* over time as:

$I\left( t \right)- I\left( t-1 \right)= aI\left( t-1 \right) + bM(t-1)$

where

- *I(t)* = number of informed individuals at time *t*,
- *a* = intrinsic sharing rate (i.e., the number of new informed individuals generated by one informed individual in one year),
- *b* = the number of new informed individuals generated per USD spent, and
- *M(t)* = the monetary investment at time *t*.

This model assumes that each informed person can generate *a·I(t)* new informed individuals through interpersonal or ICT-based sharing. In the absence of campaign funding (*M(t) = 0*), *I(t)* increases exponentially and does not naturally approach saturation.

**Model 2: Susceptible–Informed (SI) Interaction Model**

To represent saturation effects and population limits, we extended the model to include a *susceptible* population *S(t)*—individuals interested in the new technique but not yet informed. The governing equation becomes:

$I\left( t \right)- I\left( t-1 \right)= aS\left( t-1 \right)I\left( t-1 \right) + bM(t-1)$

where *S(t-1)* = number of susceptible individuals at time *t-1*.
Here, *a* represents the probability that an interaction between a susceptible and an informed individual results in conversion (i.e., *S → I*). As the number of susceptible individuals decreases, the growth in *I(t)* slows, leading to a realistic steady-state saturation.

**Model Calibration and Equivalence**

We ran simulations for both models under conditions with no external funding (*M(t) = 0*) to represent purely organic sharing. The objective was to identify a value of *a* in the SI model that produced a trajectory of *I(t)* comparable to that generated by the first (simpler) model with a defined sharing rate.

Because the first model grows exponentially, we applied a smoothing spline function to cap *I(t)* at a plausible upper limit (the total target population). We then iteratively adjusted *a* in the SI model until both models produced comparable growth trajectories.

**Results and Derived Conversion Rate**

Simulations showed that a sharing rate of 1 (i.e., each informed person generates one new informed person per year) in the first model corresponds to a = 0.0001 in the SI model - that is, one new informed individual per 10,000 interactions between susceptible and informed individuals. The time required to inform 100% of the population was highly consistent between the two models (6.55 years for Model 1 and 6.0 years for Model 2, see Figure 1).


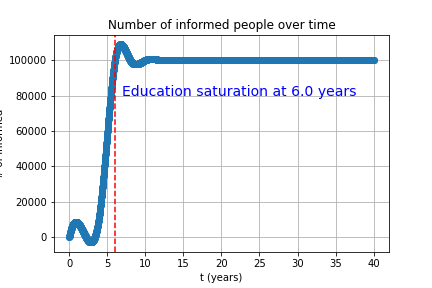

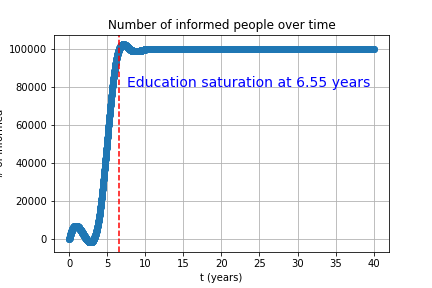


Model 2

Model 1

**Figure 1.** Predicted number of informed individuals (y-axis) over time for Model 1 (simple linear growth model) and Model 2 (susceptible–informed interaction model). The total number of informed individuals in Model 1 was capped at 100,000 to constrain unbounded exponential growth. A spline function was applied to both models to smooth the trajectories and interpolate values between yearly estimates. The trajectories align closely when a = 1 in Model 1 and a = 0.0001 in Model 2.

**Final Parameterization**

Based on this equivalence, a values for different sharing rates were calculated by dividing the sharing rate by 10,000. For example, if each informed individual shares the content with two new individuals per year, then a = 0.0002. This conversion approach was subsequently used to model dissemination scenarios based on ICT-mediated sharing rates reported in the literature.
