# Supplementary material for: Investment modeling for scalable agricultural learning
Source: PLoS One. 2026 Mar 10;21(3):e0343613. doi: 10.1371/journal.pone.0343613 (PMC12974861; doi:10.1371/journal.pone.0343613)
Supplement: Attachment — Submitted filename: PONE-D-25-41099-Note.pdf [file pone.0343613.s002.pdf]

Administrative

Manuscript ID	PONE-D-25-41099
Title	Investment Modeling for Scalable Agricultural Learning
Review round	× **Initial** ☐ Rebuttal ☐ Revision
Reviewer expertise	☐ Expert ☐ Knowledgeable × **Limited**
Reviewer	Enliang Li

---

1. Objective Summary

Authors propose a framework to test the feasibility of digital, multilingual farmer training programs economically. They review related works and utilize their own system modeling approach trying to quantify economic impacts and to help future researchers to determine if it is feasible to start a digital farmer training program.

---

2. Key contributions

1. Analyze and recognize key control components for evaluating such programs.
2. Clearly point out the problems they want to solve and define the scenarios.
3. Correct and sound mathematical deductions during the model's set-up section
4. Comprehensive experiments with analysis.

---

3. Overall evaluation

Criterion	Score[1]	Notes (1-2 lines)
**Originality / significance**	3	Assessing economic impact and cost-effectiveness of digital multilingual video contents isn't new, but its application specifically on farmers trainning could be interested.
**Technical correctness**	4	Good Math deductions.
**Empirical rigor**	3	Need more citations to cross-reference for the parameters used in the equation.
**Clarity & organization**	5	Very well organized.
**Relation to prior work**	2	Missing multi-reference to equations parameters.
**Reproducibility (code/data)**	4	Data is based on public available dataset/papers/
**Ethical impacts**	N/A	Not related.

[1]*Use the venue's scale: 1-5 as 1 is least confidence and 5 is most confidence on specific criterion.*

---

4. Recommendation

☐ Strong accept ☐ Accept × **Weak accept / borderline** ☐ Weak reject ☐ Reject

Confidence: ☐ High × **Medium** ☐ Low

The paper is well organized and delivers clear ideas on how formal system-modeling is built. However, it seems too simple, or too application-based to me as the innovation is purely about the farmers' videos it relates to.

---

5. Detailed comments to authors

5.1 Strengths:

1. Analyze and recognize key control components for evaluating such programs.
2. Clearly point out the problems they want to solve and define the scenarios.
3. Correct and sound mathematical deductions during the model's set-up section.
4. Comprehensive experiments with discussion and conclusion.

5.2 Major concerns

1. **Methodology**: Consider using a hybrid systems model instead of purely SI approach. The SI approach could be a sub-component and think about how to integrates it with cost-benefit dynamics, various groups of farmers (with different backgrounds) and time-varying decay.
2. **Writing & Presentation**: Consider better highlighting Subtitles like "Adoption base model" to improve readibility.
3. **Experiment & Observations**: Concerns raised on equations' parameters in Table 1, some key parameters like SAWBO animation and YouTube cost per informed, only citing one paper may cause bias.

5.3 Minor comments & suggestions

Line 170, "indicate" typesetting or typo issues.

5.3 Requests for clarification

The paper ignores any social and policy-related factors and only focuses on technical modeling. Is that by deliberation?

6. Recommended Reference

N/A
