# Supplementary material for: Investment modeling for scalable agricultural learning
Source: PLoS One. 2026 Mar 10;21(3):e0343613. doi: 10.1371/journal.pone.0343613 (PMC12974861; doi:10.1371/journal.pone.0343613)
Supplement: Attachment — Submitted filename: 2. Response to Reviewers pr8_rp_Nov_12_2025.docx [file pone.0343613.s004.docx]

**PlosOne**

**Point-by-Point Response to Reviewers**

PONE-D-25-41099

Title: Investment Modeling for Scalable Agricultural Learning

Dear editor and reviewers,

We sincerely appreciate your thoughtful comments. In response to your feedback, we have made several changes to the paper, which are outlined in detail in the point-by-point response below. Our comments are in *italics*. Parts taken from the paper are in "quotation marks", and new sections added are "underlined".

Reviewer #1:

1. Lack of Heterogeneity and Parameter Correlation
The model assumes that key parameters such as adoption rates, income gains, and campaign costs are fixed and independent across contexts. However, in practice, these factors are often highly heterogeneous and interdependent. For example, adoption rates vary widely depending on farmers’ literacy levels, cultural norms, prior exposure to digital tools, and socioeconomic conditions. Similarly, income gains differ substantially by crop type, farming system, and baseline productivity. Furthermore, multilingual adaptation does not guarantee equal comprehension — regional dialects, education levels, and digital literacy gaps often affect knowledge transfer effectiveness. Ignoring such heterogeneity and parameter correlations risks biasing ROI estimates and overstating scalability. I recommend that the authors expand the discussion to clarify how these correlations may impact overall findings.

*We thank the reviewer for this thoughtful comment.*

*Heterogeneity was addressed in our study through sensitivity analyses conducted over large parameter ranges, which enabled us to identify the parameters most strongly influencing ROI. To clarify this, we added the following to the methods section:*

*"The base model assumes fixed parameters, yet farming contexts and populations are inherently diverse. In reality, factors such as literacy levels, cultural norms, prior exposure and access to digital tools, crop type, and baseline productivity can all lead to substantial variation in outcomes. To account for this heterogeneity, we conducted simulations across a wide range of parameter values to identify the contextual conditions under which positive returns are most likely to occur. Simulations varied system inputs (i.e., production cost, deployment cost, adoption cost) and parameters (i.e., sharing rate, adoption rate, annual income, income gain) by 1/4 x, 1/2 x, 2 x, and 4 x of their base model values to evaluate their impact on overall returns. In addition to IRR, the expected overall cost and revenue over the 3-year period, time to inform 10% of the population, and number of adopters were also estimated."*

*We acknowledge that correlations among parameters were not explicitly modeled. The strength and structure of these correlations are not yet well understood, but this is an important area for future research that will benefit from real-world data collection to better assess parameter interdependencies. We acknowledge this limitation and emphasize the need for incorporating parameter correlations in subsequent work:*

*"In terms of limitations, our study permitted system parameters to vary independently in simulations, whereas in reality, these attributes are likely correlated. For instance, adoption costs and adoption rates likely exhibit interdependence. By modelling more specific scenarios, it should be possible to account for these dependencies and to generate more precise estimates. Subsequent research efforts should prioritize real-world data collection to refine models, while leveraging current model insights."*

*In addition, we have made the original source code available for readers interested in running their own simulations. The code has been updated to explore how parameter interdependencies impacts ROI. While we considered adding additional simulation results to the manuscript, we felt that current knowledge about these dependencies is too limited to justify detailed analysis, and including extensive new outputs would have lengthened the paper without substantially improving interpretability.*

2. Writing Quality and Clarity
The manuscript would benefit from improved English writing to enhance clarity, especially in the methodology and results sections. Certain assumptions and parameter derivations are difficult to follow, which may limit reproducibility and interpretability. Providing clearer explanations, illustrative tables, and stronger linkage between model assumptions and referenced literature would significantly improve readability. Prior PLOS ONE studies on agricultural investment modeling demonstrate the value of transparent parameter reporting and plain-language summaries when communicating findings to broader audiences.

*We thank the reviewer for highlighting the importance of clarity and transparency in communicating methodology and results. We strengthened the* ***connections between assumptions and supporting literature*** *throughout the revised text.* ***Table 1*** *presents the base model values alongside corresponding estimates from the literature, to make parameter choices more transparent.*

*In addition, we expanded the explanation of the* ***sharing rate derivation*** *and provided more details in the* ***supplemental material****. We found that incorporating the complete derivation into the main text disrupted readability, but the supplemental file now contains the necessary information to ensure reproducibility. The following was modified in the methods section:*

*"Obtaining an initial estimate of a for the SI model is not straightforward (see supplemental material for details). We estimated a, the probability that an interaction between a susceptible and an informed individual results in a new informed individual, to be approximately 0.0001, equivalent to one new informed person per 10,000 such interactions. For ICT-based dissemination, the estimated number of shares was approximately four for every informed person each year[7]. Accordingly, a increases to 0.0004 (4 × 0.0001)."*

*Also, we revised the* ***Methodology*** *section to clarify the derivation for the adoption rate.*

*"Not all informed people will adopt the technique in the learning initiative; therefore, another parameter was included reflecting the adoption rate. For the base model, an adoption rate of 0.055 is used. This rate is derived from the reported average adoption rate of 49.8% for in-person training[32], adjusted to account for an estimated 11% knowledge uptake and adoption when transitioning from in-person training to mass dissemination methods (49.8% × 11% ≈ 5.5% or 0.055) [33] (Table 1)."*

*Finally, on the point of reproducibility, as mentioned in the first response, the code is now fully available to ensure transparent and replicable results.*

3. Lack of Sensitivity and Robustness Analysis
The reported 30-year IRR of 657% is extraordinarily high compared to typical ex-post evaluations of agricultural extension programs, which often report returns closer to 30–50%. While the authors attribute this to cost efficiencies enabled by digital dissemination, the manuscript lacks sufficient sensitivity analyses and robustness checks to support this conclusion. For example, how would the IRR change if adoption rates were lower, income gains were overestimated, or translation and campaign costs were higher? Including formal sensitivity testing or Monte Carlo simulations would greatly improve the credibility and policy relevance of the results.

*We thank the reviewer for this important observation. Sensitivity analysis was a central focus of the study. We systematically perturbed key parameters (including adoption rates, income gains, and campaign costs) across a wide range of values. Our intention in varying one parameter at a time was to provide readers with a clear understanding of the isolated effect of each assumption on ROI (e.g., showing how doubling or halving a parameter value shifts projected returns).*

*We agree with the reviewer that additional robustness checks, such as Monte Carlo simulations, could provide further insight by allowing multiple parameters to vary simultaneously according to specified probability distributions. At present, we do not have empirical information on the underlying distributions of many parameters. This limitation is the main reason we chose the proposed approach, which applies a "theoretical" stress to the model to simulate sensitivity scenarios based on the limited (as we acknowledged) evidence currently available. However, we agree that Monte Carlo approaches have merit, and we plan to incorporate them in future work as more real-world data on parameter distributions become available.*

*Regarding the claim that cost efficiencies enabled by digital dissemination drive the high ROI, we agree that this remains speculative at this stage. We have therefore softened the assertion in the manuscript to reflect this uncertainty.*

*"Our study examined a broad range of scenarios, with the base model projecting an internal rate of return (IRR) of 208% for a target population of 100,000. Notably, our 30 year IRR estimate of 657% far exceeds the reported 30 year average of 45% for agricultural learning initiatives in Africa[34]. While this discrepancy raises questions about model accuracy, it may also reflect fundamental differences in dissemination approaches. Published returns are largely derived from farmer field schools, which involve deployment costs ranging from $9 to $35 per farmer[25, 26]. In contrast, our study focuses on digital learning, which offers greater cost efficiency (our base model assumes a cost of $1.80 per farmer) potentially driving higher returns. A follow-up simulation using a $35 per new informed farmer resulted in a 30-year return of 52%, aligning more closely with published estimates. Nevertheless, given the preliminary nature of our analysis, we cannot state with certainty that digital dissemination yields returns as high as those simulated, nor can we definitively identify the key drivers of these elevated returns if they do exist."*

Reviewer #2:

Strengths:
1. Analyze and recognize key control components for evaluating such programs.
2. Clearly point out the problems they want to solve and define the scenarios.
3. Correct and sound mathematical deductions during the model’s set-up section.
4. Comprehensive experiments with discussion and conclusion.

*We thank the reviewer for recognizing these strengths in our work.*

Major concerns
1. Methodology: Consider using a hybrid systems model instead of purely SI approach. The SI approach could be a sub-component and think about how to integrates it with cost-benefit dynamics, various groups of farmers (with different backgrounds) and time-varying decay.

*We appreciate the reviewer’s suggestion to move toward hybrid models that incorporate diffusion dynamics, cost–benefit interactions, population heterogeneity, and time-varying decay. Our current study intentionally employed a simplified framework to preserve tractability and to align with systems identification principles, which we plan to apply more fully as real-world data become available. In this approach, parameters will be treated as time-varying coefficients that can be re-estimated to capture shifts in system dynamics (e.g., rising costs when reaching marginal groups), rather than assumed to be fixed. This allows the use of linear system theory tools for parameter identification and the design of feedback mechanisms to optimize educational campaigns. We view the present model as a foundation that can be iteratively improved as datasets are collected, including expanding to multiple models to represent more homogeneous farmer subpopulations. Time-varying decay will similarly be addressed through adaptive modeling, where parameter estimates are updated with observed data rather than relying on assumed decay rates.*

2. Writing & Presentation: Consider better highlighting Subtitles like “Adoption base model” to improve readibility.

*We are unsure how to interpret this comment, as the subtitles are already formatted in bold, 16-point font, which distinguishes them from the main text. If we have misunderstood the reviewer’s concern, we apologize and would welcome clarification.*

3. Experiment & Observations: Concerns raised on equations’ parameters in Table 1, some key parameters like SAWBO animation and YouTube cost per informed, only citing one paper may cause bias.

*We acknowledge the reviewer’s concern regarding potential bias due to limited citation of cost parameters. To our knowledge, no peer-reviewed publications report the cost per new informed individual for agricultural YouTube-based dissemination campaigns. However, we have conducted several such campaigns in multiple regions and observed costs ranging from US $0.10 to US $5.20 per new informed individual. Taking the mean across campaigns yields an estimated US $1.80 per new informed, which was used in the model. To enhance transparency, we updated Table 1 to report both the mean and the range of costs derived from these types of campaigns.*

*"$1.80 per new informed using SAWBO animation and YouTube[8] (range = $0.10 to $5.20 (unpublished); $9 to $35 per farmer for farmers field school[30, 31]"*

Minor comments & suggestions
Line 170, “indicate” typesetting or typo issues.

*We carefully reviewed the manuscript for all occurrences of “indicate” and “indicates.” Each usage appears to be grammatically correct.*

Requests for clarification
The paper ignores any social and policy-related factors and only focuses on technical modeling. Is that by deliberation?

*The focus of this paper was intentionally placed on the technical modeling component, serving as a proof-of-concept for applying system modeling to evaluate the economic viability of ICT-based agricultural learning. Social and policy-related factors influencing agricultural learning initiatives are complex and beyond the scope of the present study. To explore policy-related factors, we are developing a separate paper that employs real-world data to simulate the effects of language inclusion policies. Simulations illustrate trade-offs between language inclusivity and overall program reach, which vary across regions. We believe that maintaining a clear technical focus in the current paper provides a strong foundation for subsequent research integrating these broader social and policy contexts.*

Reviewer #3:

The manuscript presents a framework for evaluating the economic impact of scalable agricultural learning initiatives.

While the manuscript applies a systems modeling framework, it lacks comprehensive coverage of recent developments in this area. More recent applications of system approaches could be discussed to highlight what is novel in this work.

*We thank the reviewer for this helpful suggestion. In response, we have expanded the Introduction to include a discussion of prior system dynamics applications in agriculture and natural resource management. We also highlight recent calls for next-generation agricultural system models that leverage advances in ICT. These additions provide broader context and demonstrate how our work builds on and extends this body of research.*

*"The primary goal of this study is to develop a general framework for evaluating the economics of learning initiatives using a systems approach. Our work builds on a growing body of system-based applications in agriculture and natural resource (AGNR) management. Prior studies have emphasized the value of system dynamics for addressing complex AGNR problems, particularly in identifying trade-offs between short- and long-term strategies[20]. Other work has demonstrated the utility of systems modeling for evaluating technology adoption and diffusion processes in agribusiness[21], assessed policy instruments such as payment for environmental services in livestock systems[22], and developed integrated agroecosystem models that capture environmental, economic, and social trade-offs[23]. More relevant, the need for next-generation agricultural system models that leverage advances in ICT to improve decision-making and impact assessment has also been highlighted[24]. Together, these studies underscore the versatility of systems approaches. The present study is intended as a preliminary exploration to generate early insights into the key drivers that enhance returns in Research-for-Development (R4D) learning initiatives. It represents a foundational step in an iterative process that includes the design of future data collection efforts to improve model accuracy and the use of refined models to identify and prioritize high-impact agricultural learning strategies."*

20.	Turner BL, Menendez HM, Gates R, Tedeschi LO, Atzori AS. System Dynamics Modeling for Agricultural and Natural Resource Management Issues: Review of Some Past Cases and Forecasting Future Roles. Resources. 2016;5(40). doi: 10.3390/resources5040040.

21.	Fisher D, Norvell J, Sonka S, Nelson M. Understanding technology adoption through system dynamics modeling: Implications for agribusiness management. International Food and Agribusiness Management Review. 2000;3. doi: 10.1016/S1096-7508(01)00048-9.

22.	Benavides RAM, Gaona RC, Atzori AS, Sánchez LF, Guerrero HS. Application of a system dynamics model to evaluate the implementation of payment for environmental services as a reconversion mechanism in high mountain farming. Ecological Modelling. 2023;484. doi: 10.1016/j.ecolmodel.2023.110469.

23.	Papanicolaou AN, Basnet K, O’Brien PL, Wacha KM, Malone RW, Archer DW. A system dynamics modeling framework to evaluate impacts on economic, environmental, and social quality components of a U.S. Midwestern agroecosystem transitioning from row crop agriculture to mixed farming systems. Ecological Modelling. 2025;506. doi: 10.1016/j.ecolmodel.2025.111142.

24.	Antle JM, Jones JW, Rosenzweig CE. Next generation agricultural system data, models and knowledge products: Introduction. Agric Syst. 2017;155:186–90. doi: 10.1016/j.agsy.2016.09.003. PubMed PMID: 28701811; PubMed Central PMCID: PMCPMC5485643.

The model used in the article relies on digital media, as part of the broader ICT landscape. While systemic approaches have seen limited application in this specific context, we believe that applying a systemic lens specifically to digital media represents a valuable contribution to the existing literature on systems modeling. Our goal in this initial work was to prioritize simplicity and tractability, which will be important in the future when model parameters are estimated through system identification methods and educational campaign effectiveness is enhanced using optimization approaches. If future analyses indicate that a more explicit representation of digital media dynamics is required to improve model accuracy or predictive performance, such extensions will be incorporated. This perspective is reflected in the manuscript, where we note that future research may explore more complex frameworks (e.g., social network graphs or time-varying models) as data availability and model maturity increase:

*"Furthermore, future research should explore alternative model types, such as social network graphs and time-varying models, to enhance predictive capabilities while upholding reliability and interpretability. As more granular data becomes available, models can evolve in complexity as needed to better mirror real-world intricacies."*

Further in response to the reviewer's comment, we have expanded the Conclusion section to clarify how digital media attributes reinforce the model’s findings and are partially captured through its structure. Specifically, we highlight:

- **Multilingual adaptation:** digital formats enable rapid translation and distribution across diverse linguistic groups.
- **Persistence and reusability:** digital content can be reused and shared over time at minimal cost, unlike in-person training.
- **Network effects:** dissemination is amplified through peer-to-peer sharing and social platforms, dynamics that are represented in our SI-inspired modeling framework.

The revised paragraph in the Conclusion now reads:

*"In regions like Africa, where rapid population growth and climate change threaten food security, the urgency to scale and optimize agricultural learning is especially acute. As our simulations suggest, ICT-based approaches hold significant promise, not only for combating hunger, but also for advancing economic development among farming communities. Moreover, digital media offers several unique advantages over in-person training that reinforce this potential. Its flexibility enables rapid multilingual adaptation and dissemination across diverse linguistic and cultural contexts; its persistence allows content to be reused over time at minimal cost; and its network effects arising from peer-to-peer sharing and social platforms further amplify the reach of digital learning, an effect captured in our SI-inspired modeling framework. Collectively, these characteristics highlight the potential of ICT-based learning to complement traditional agricultural capacity-building approaches by promoting more inclusive, efficient, and sustainable forms of knowledge diffusion in the Global South."*
